# Amputation in the Prairies

**Published:** 1845-03

**Authors:** 


*Amputation in the Prairies.*—The following case of successful amputation on one of our western wilds, has been going the rounds of the journals; we insert it here, as it furnishes another to the many evidences which we already possess. of the great resources of nature, which, under the most adverse circumstances, is capable of accomplishing so many unexpected and surprising results:

"A few days before the caravan had reached Walnut Creek, a Mr. Broadus, in attempting to draw his rifle from a wagon, muzzle foremost, discharged its contents into his arm. The bone being dreadfully shattered, the unfortunate man was advised to submit to an amputation at once; otherwise, it being in the month of August, and excessively warm, mortification would soon ensue. But Broadus obstinately refused to consent to this course, till death began to stare him in the face. By this time, however, the whole arm had become gangrened, some spots having already appeared above the place where the operation should have been performed. The invalid's case was therefore considered perfectly hopeless, and he was given up by all his comrades, who thought of little less than to consign him to the grave. But being unwilling to resign himself to the fate which appeared frowning over him, without a last effort, he obtained the consent of two or three of the party, who undertook to amputate his arm, merely to gratify the wishes of the dying man, for in such a light they viewed him. The only 'case of instruments' consisted of a handsaw, a butcher's knife, and a large iron bolt. The teeth of the saw being considered too coarse, they went to work and soon had a set of fine teeth filed on the back. The knife having been whetted keen, and the bolt laid upon the fire, they commenced the operation; and in less time than it takes to tell it, the arm was opened round to the bone, which was almost in an instant sawed off, and with the whizzing hot iron, the whole stump was so effectually seared as to close the arteries completely. Bandages were now applied, and the company proceeded on their journey as though nothing had occurred. The arm commenced healing rapidly, and in a few weeks the patient was sound and well, and is perhaps still living to bear witness to the superiority of the 'hot-iron' over ligatures in 'taking up arteries.'"—*Gregg's Commerce of the Prairies.*

